# Organosilicone Compounds in Supercritical Carbon Dioxide

**DOI:** 10.3390/polym14122367

**Published:** 2022-06-11

**Authors:** Victor E. Sizov, Vadim V. Zefirov, Marat O. Gallyamov, Aziz M. Muzafarov

**Affiliations:** 1 Faculty of Physics, Lomonosov Moscow State University, 119991 Moscow, Russia; glm@spm.phys.msu.ru; 2 Enikolopov Institute of Synthetic Polymeric Materials, Russian Academy of Sciences, 117393 Moscow, Russia; aziz@ispm.ru; 3 Nesmeyanov Institute of Organoelement Compounds, Russian Academy of Sciences, 119991 Moscow, Russia; vv.zefirov@physics.msu.ru

**Keywords:** polydimethylsiloxane, silicone rubbers, supercritical fluid, CO_2_

## Abstract

This review considers the key advantages of using supercritical carbon dioxide as a solvent for systems with organosilicon compounds. Organosilicon polymeric materials synthesis as well as the creation and modification of composites based on them are discussed. Polydimethylsiloxane and analogues used as polymerization stabilizers and nucleation promoters in pore formation processes are analyzed as well.

## 1. Introduction

Supercritical CO_2_ (scCO_2_) is an alternative promising solvent that has been actively used in recent decades to simplify many processes of polymer synthesis, modification, decomposition, etc. [1]. CO_2_ is a gas under normal conditions, which allows it to be effectively removed from the resulting material and thus solves the problem of residual solvent. The viscosity of scCO_2_ is rather low and approaches the viscosity of gases when the pressure decreases. The small size of CO_2_ molecules (0.4 nm) provides high coefficients of self-diffusion (≈10^−4^ cm^2^ s^−1^) and diffusion into various materials (≈10^−7^ cm^2^ s^−1^), which are two orders of magnitude higher than those of organic solvents. It should be noted that the solubility parameters of certain materials in scCO_2_ can be varied by changing the thermodynamic parameters of the medium (temperature, pressure, and density).

At present, supercritical CO_2_ has a wide range of applications. A large number of works have been devoted to the synthesis of various polymer particles in supercritical fluids, including CO_2_ [2], the creation of composites based on compounds soluble in CO_2_ and insoluble substrates [3], the modification of polymers, the synthesis of catalysts, polymerization, and polycondensation [4,5]. One more significant field is organosilicon polymers, which, along with fluoropolymers, form two large classes of macromolecular compounds soluble in scCO_2_ [6,7].

The purpose of this review is to describe the current level of application of supercritical carbon dioxide for organosilicon systems, including a discussion of actual development trends and the identification of still insufficiently developed but promising research areas.

We focused on the results of (co)polymerization in scCO_2_, the advantages of using organosilicon compounds as polymerization stabilizers, and the development of approaches to coating from carbon dioxide. In this review, the authors tried to supplement the previous review on related topics published in 2016, where the role of supercritical carbon dioxide in various chemical processes both as a solvent and a reagent was analyzed [5]. In the present review, we focus on recently published articles as well as those not previously reviewed in Ref. [5].

## 2. Synthesis in scCO_2_

### 2.1. Benefits of scCO_2_

Supercritical CO_2_ is already a common medium for laboratory synthesis, including the one of organosilicon compounds. The high diffusion rate and the absence of capillary effects significantly accelerate the kinetics of reactions in supercritical fluids as compared with the usage of typical liquid solvents. This is also due to the significantly lower viscosity of the supercritical fluid and, as a consequence, of the solutions of organosilicon compounds in it. Since CO_2_ is a chemically inert and harmless gas at normal pressure, there is no residual solvent problem. On the other hand, the dissolving power of scCO_2_ can be easily varied by tuning thermodynamic parameters of the system (temperature and pressure). This opens up the possibility of extracting and separating residual monomers and catalysts for subsequent reuse. Due to that, the method can be applied to a wide range of systems. 

### 2.2. Obtaining Aerogels in scCO_2_

In 1997, Loy et al. described the first one-step sol–gel polycondensation of tetraalkoxysilanes or 1,4-bis-(triethoxysilyl)benzene (BESP) in scCO_2_: a mixture of alkoxysilanes or BESP and anhydrous formic acid was placed in a reactor, where scCO_2_ was applied. The system was exposed for 12 h and slowly decompressed, this way transforming scCO_2_ to a gaseous state and forming monolithic silica or polysilsesquioxane aerogel [8]. The use of scCO_2_ for carrying out such condensation reactions remains an urgent problem. Freed from the solvent in the absence of capillary forces, aerogels retain most of their original volume, which allows them to be effectively used as heat insulators, catalysts, etc. However, typically, low-molecular-weight liquid by-products of the condensation reaction, such as formic acid esters, require careful removal before the decompression procedure to prevent the retrograde condensation in the pores of the formed aerogel (the retrograde condensation is a counterintuitive phenomenon of liquid droplets formation in an initially homo-phase supercritical mixture during decompression) [9].

In 2015, Zou et al. demonstrated the process of obtaining elastic superhydrophobic cross-linked polydimethylsiloxane (PDMS) aerogels for water purification [10]. In the presence of Karstedt’s catalyst (divinyl-containing Pt disiloxane complexes), oligomeric vinyl-terminated dimethylsiloxane was hydrosilylated with a hydride-containing oligomer. No liquid by-products of the reaction are formed with this approach, which guarantees the absence of possible complicating phenomena, such as the retrograde condensation at the stage of removal of the supercritical solvent. Various aerogel densities obtained by using diverse initial siloxane reagents and variations in their concentrations ranged from 150 to 260 mg cm^−3^. The method is fast in comparison with the classical schemes for obtaining aerogels, especially those involving a stage of solvent replacement, and it requires only a few hours.

In 2017, our group applied a similar hydrosilylation method for the synthesis of organosilicone aerogels in scCO_2_ [11]. Commercially available hydride-containing functionalized PDMS and a wide range of divinyl PDMS oligomers of variable length were used as reagents. Speier’s catalyst (chloroplatinic acid) or scCO_2_-soluble organometallic compound (1,5-cyclooctadiene)dimethylplatinum (PtMe_2_(COD)) were chosen as catalysts among a wide range of compounds. It was shown that the properties of the resulting aerogels are primarily affected by the density of CO_2_ and the length of the divinyl cross-links from PDMS units. By varying these parameters and the concentration of reagents, it was possible to obtain either superhydrophobic monolithic aerogels with a density of down to ~130 mg cm^−3^, which is slightly lower than the values achieved in the previous work, or microgranules with a diameter of ~0.5 mm. It is important to emphasize that the use of supercritical fluid soluble catalysts opens the potential for their extraction, at least partial, separation and reuse, which is an important problem in conventional synthesis in the presence of liquid solvents.

In subsequent work, a wider range of vinyl- and hydride-containing siloxane precursors was used, and the mechanical properties of aerogels obtained in scCO_2_ were studied depending on the nature of the reagents and their quantitative ratio [12]. When hyperbranched silsesquioxane hydrides are used, the Young’s modulus of the resulting aerogels is down to 3.6 kPa. On the other hand, the use of tetraallylsilane with oligomeric linear hydride-containing siloxane makes it possible to increase the Young’s modulus to 20–40 kPa and higher, depending on the ratios of the initial compounds during loading.

Mahadik et al. proposed a modified method for the preparation of aerogels from tetramethylorthosilicate (TMOS), including in combination with trimethoxymethylsilane in methanol [13]. In contrast to the well-known model, when the sol is heated slowly during synthesis in order to avoid a temperature gradient, it was proposed to provide fast heating. This approach allows for a rapid transition of methanol to the supercritical state so that gelation proceeds after this transition. For this, nitrogen was preliminarily pumped to the reactor at a partial pressure of above 50 bar, which increased the boiling point of methanol. The authors claim that this approach makes it possible to transfer methanol directly to the supercritical state, bypassing phase separation and thereby eliminating capillary effects. As a consequence, aerogels with minimal shrinkage and high specific surface area can be rapidly formed. The density of the resulting aerogels, depending on the concentrations and the combination of reagents, ranged from 35 to 170 mg cm^−3^.

It is worth discussing the success of classical processes for the synthesis of aerogels at atmospheric pressure followed by supercritical drying of the material. Thus, in 2007, the process of obtaining elastic aerogels based on methyltrimethoxysilane was described using the classical approach of supercritical drying [14,15]. Their flexible behavior is due to the silsesquioxane nature: the density of the network formed is lower than that in silica. At the same time, a high concentration of hydrophobic methyl groups, as well as a lower content of residual silanol groups, ensures the reversibility of deformations. Moreover, due to the excellent mechanical properties, it was also possible to obtain methyltrimethoxysilane-based xerogels by a simple conventional drying procedure.

A milestone on the way to obtaining aerogels with high mechanical properties was the series of works by Nakanishi et al. [16,17,18]. Transparent aerogels based on polyethylsilsesquioxane (PESQ, CH_3_CH_2_SiO_1.5_) and polyvinylsilsesquioxane (PVSQ, CH_2_=CHSiO_1.5_) with high thermal insulation properties were obtained. In addition, it was possible to carry out additional parallel cross-linking (according to the free radical mechanism, along with hydrolysis/condensation) in the resulting PVSQ, which provided an additional increase in mechanical characteristics. It is important that due to the flexibility and strength of the formed gels, it turned out to be possible to create xerogels that are easy to obtain and similar in properties. In order to further improve the properties of thermal insulation and flexibility, aerogels based on polyvinylpolymethylsiloxane, polyallylpolymethylsiloxane, and other compounds were obtained [16,17,18].

Due to the technological complexity of obtaining aerogels, maximum optimization of each stage is needed. In 2021, a highly efficient method for obtaining siloxane aerogels was described: the gelation time (the first stage of the process) was reduced to 5 min (subsequent drying of the gel was carried out in scCO_2_) [19]. During the classical aerogel-obtaining method, the hydrolysis stage proceeds much faster than condensation in acid catalysis, while the situation is reversed in basic catalysis. Therefore, a combined approach is often used: acid catalysis followed by basic catalysis. However, this method does not always allow simplifying the process and reducing the time. Therefore, it was proposed to use an “amphoteric” catalyst, which would be widespread, cheap and sufficiently active. Thus, the synthesis was carried out at room temperature and atmospheric pressure, BF_3_·Et_2_O was used as the desired catalyst, and acetone was used as a solvent. Great work has been carried out to analyze the influence of various parameters, including the type of precursor, type of solvent, and concentration of components, on the morphology of the obtained samples. For example, the specific surface area of aerogels obtained from TMOS varied in the range from 800 to 1200 m^2^ g^−1^, while Young’s modulus varied from 0.1 to 6 MPa.

The wide applications potential of organosilicon aerogels and the problems, primarily related to the complexity of the synthesis process and the mechanical stability of materials [20], stimulate further study of their synthesis.

### 2.3. Copolymers Based on Organosilicon Compounds in scCO_2_

The solubility of various classes of monomeric compounds and the high diffusion rate in scCO_2_ make it a promising medium for copolymerization reactions compared to organic solvents. Although the processes of copolymerization in scCO_2_ medium in general have been known for a relatively long time and are well systematized [21], not much work has been conducted on the synthesis of copolymers of organosilicon compounds.

**Figure 1:**
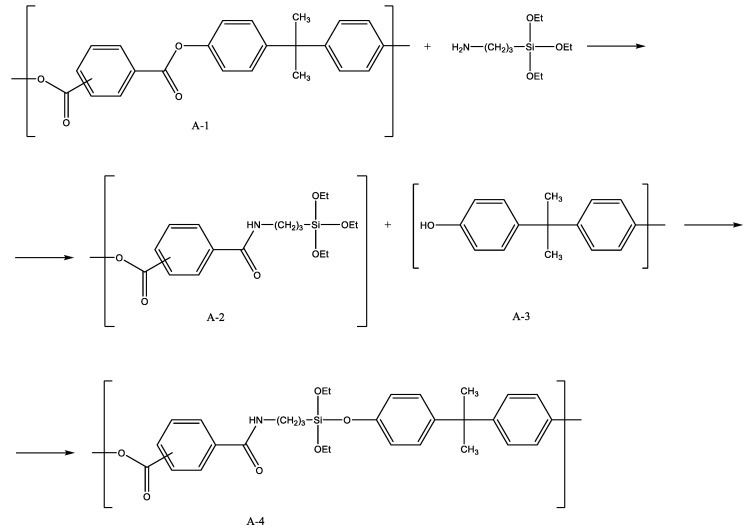
Scheme for the synthesis of poly(acrylate-siloxane). Adopted from Ref. [22].

The synthesis of a poly(acrylate-siloxane) copolymer in supercritical carbon dioxide was proposed (Figure 1) [22]. The reaction of poly(4,4′-isopropylidene-2,2′-diphenylene (tere) isophthalate) and 3-aminopropyltriethoxysilane was carried out in scCO_2_ at 150 bar and 100 °C. Under such conditions, aminolysis of ester bonds in the initial polymer occurred. This led to the formation of amides containing ethoxy groups and their subsequent condensation with the second chain fragment (by the reaction of ethoxy groups and terminal phenolic groups), to poly(acrylate-siloxane). The silane reagent incorporation into the structure of macromolecules made it possible to significantly improve the thermomechanical properties of the initial polymer. It is worth noting that ethoxy groups on silicon atoms remain reactive. This leads to the formation of an insoluble in standard solvents material upon exposure to air for a month. The long-term stability of Si–O–C bonds in the aromatic structural fragments of the polymer requires separate consideration.

**Figure 2:**
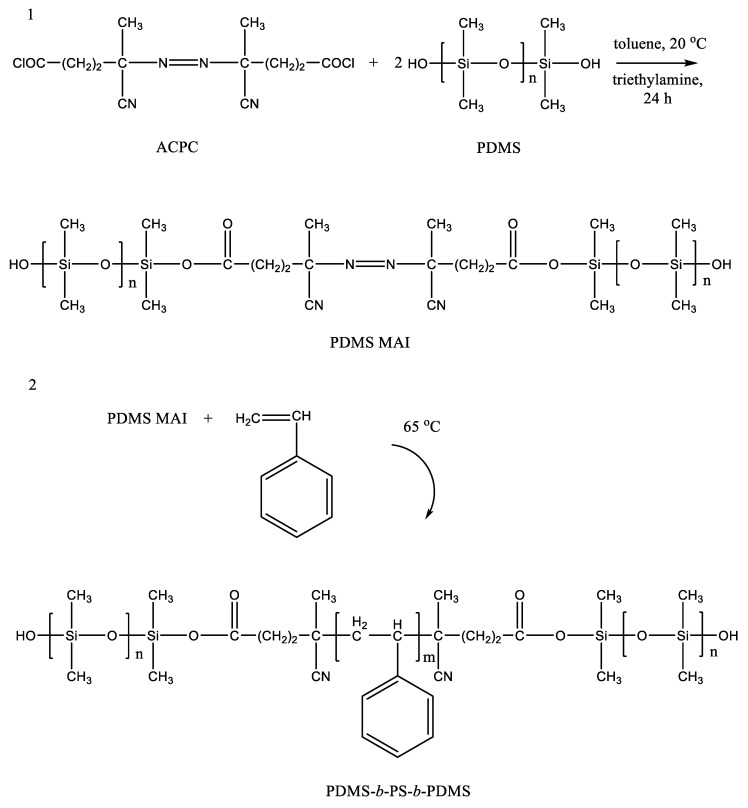
Stages of synthesis of the macroinitiator PDMS MAI (**1**) and triblock copolymer PDMS-b-PS-b-PDMS (**2**). Adopted from Ref. [23].

Studies on the synthesis of triblock copolymers based on PDMS and polystyrene are known [23]. The authors proposed a radical polymerization of styrene and PDMS macroinitiator (PDMS-MA) in scCO_2_ medium. PDMS-MA was previously obtained by the reaction of hydroxyl-terminated PDMS with 4,4-azobis(4-cyanopentanoyl chloride) (Figure 2). The bond to the silicon atom between the forming blocks is ester, which has been confirmed by the detected peak in the IR spectrum at 1720 cm^−1^. The Si–O–C group in the aromatic structural fragment is a weak link due to the unpredictability of its stability under various conditions. On the other hand, its presence may prove to be an advantage in the directed utilization of the polymer, acting as an element of programmable decomposition into separate fragments in the recycling process.

**Figure 3:**
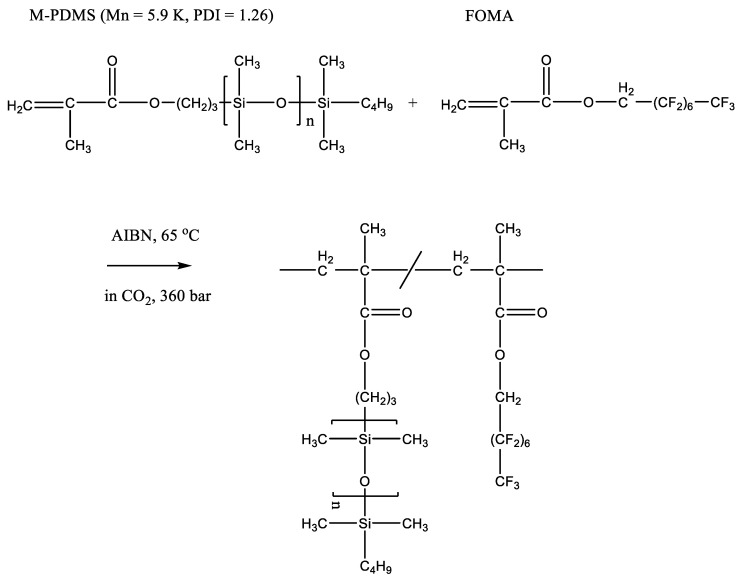
Copolymerization of PDMS and FOMA in supercritical CO_2_. Adopted from Ref. [25].

A number of other examples of radical (co)polymerization using organosilicon monomers in supercritical carbon dioxide are known. Thus, using azobisisobutyric acid dinitrile as an initiator, a copolymer based on acrylic acid and 3-[tris(trimethylsilyloxy)silyl]propyl methacrylate was synthesized [24]. The viscosity of the solutions of the obtained copolymers showed a pronounced dependence on pH. The values of the average viscosity molecular weight increased in the range of (0.56–2.4) × 10^6^ with an increase in the fraction of the organosilicon comonomer in the reaction mixture, which determined its proportion in the resulting copolymer. The authors note that the organosilicon fraction in the polymer systematically exceeded the organosilicon fraction in the reaction mixture, which indicates a higher activity of the organosilicon comonomer in comparison with acrylic acid in the reaction. Another research group investigated the copolymerization of dihydroperfluorooctyl methacrylate (FOMA) with methacryloxypropyl-terminated PDMS, M_n_ ~ 5900 kg mol^−1^ (Figure 3) [25]. The resulting copolymer turned out to be microphase separated into FOMA and PDMS domains. The GPC-measured values of M_n_ of the obtained copolymer were in the range of (230–36) kg mol^−1^ and decreased with an increase in the proportion of PDMS in the copolymer (which was determined by its fraction in the reaction mixture). The low values of the polymerization degree related to an increase in the proportion of PDMS are explained by a possible contribution of steric restrictions, a low concentration of vinyl groups in the macromonomer, as well as chain termination and transfer reactions.

### 2.4. Spatially Regular Well-Defined Mesoporous Organosilicon Materials

The absence of capillary effects and high diffusion coefficient makes scCO_2_ a superb media to obtain porous structures of complex geometric shapes.

**Figure 4:**
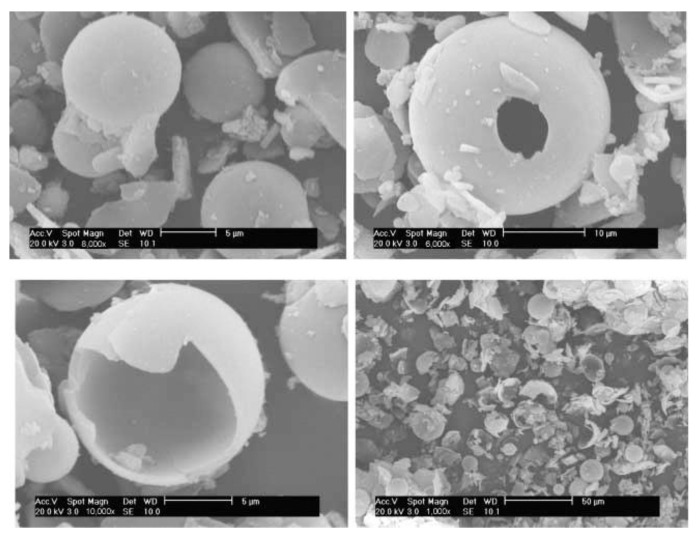
SEM images of hollow silicon dioxide spheres. Republished with permission of Royal Society of Chemistry, © 2005, from Ref. [26].

In Ref. [26], supercritical carbon dioxide was used as a medium component for the hollow organosilicon spheres synthesis. Particles with an outer diameter of ≈5–10 microns were obtained in a CO_2_ emulsion in water with the addition of a poly(ethylene oxide)-b-poly(propylene oxide)-b-poly(ethylene oxide) triblock copolymer as a surfactant and an organosilicon precursor tetraethoxysilane (TEOS) (Figure 4). The resulting spheres had poor mechanical properties, partially collapsing during decompression. Nevertheless, the fundamental possibility of creating such structures, which are promising for problems of catalysis and drug delivery systems, was demonstrated. In addition, the authors indicated the potential possibilities of varying the properties of the obtained spheres by changing the reagents and the thermodynamic parameters of the medium.

In a subsequent similar work [27], the authors also managed to obtain more complex structures, i.e., single-layer and multilayer hollow organosilicon spheres. Pluronic P123 triblock copolymer and a mixture of cationic (hexadecyltrimethylammonium bromide) and anionic (sodium dodecyl sulfate) surfactants were used as a double template for the spheres’ formation. The organosilicon precursor was 1,4-bis(triethoxysilyl)benzene. Single-layer and multilayer spheres with an average diameter of 40 to 200 nm were obtained, and varying the pressure of the medium—a mixture of the aqueous phase and compressed CO_2_—made it possible to change the wall thickness of the obtained particles.

In 2016, the same group of authors proposed a “green” method for obtaining bifunctional periodic mesoporous organosilicas in a mixture of an aqueous phase and compressed CO_2_ [28]. The resulting material had hexagonally packed cylindrical pores with a diameter of 3.2–6.5 nm, which could be controlled by changing the applied CO_2_ pressure (from 4 to 6 MPa). The same P123 copolymer and 1,4-bis(triethoxysilyl)benzene were used together with 2,5-bis(triethoxysilyl)thiophene as two organosilicon precursors in the synthesis. The authors believe that the morphology of the resulting structures is primarily determined by the kinetics of hydrolysis and condensation processes, which, in turn, depends on the pH of the aqueous phase of the synthesis medium. The authors used the bromophenol blue indicator to detect the pH values of the aqueous phase saturated with CO_2_ under pressure (formation of carbonic acid [29]) by means of spectrometry. It was concluded that at CO_2_ pressure of 3.9 MPa or less, the pH of the medium was insufficient for the rapid hydrolysis and condensation of silicon-containing precursors, which led to the formation of less regular structures. At higher pressures, it was possible to form worm-like mesoporous periodic structures, with the pore size and wall thickness increasing with an increase in CO_2_ pressure. This is due to the following factors: firstly, an increase in the concentration of dissolved CO_2_ facilitates the penetration of gas molecules into the hydrocarbon chains regions of the micelles, contributing to their volume growth; secondly, lower pH values accelerate the sol–gel reaction, which contributes to the rapid condensation of hydrolyzed siloxanes.

Furthermore, supercritical CO_2_ is used to create periodic mesoporous organosilicon films, which are required in sensors, detectors, separators, low permittivity materials and others [30]. Well-ordered mesoporous organosilicon films were prepared by the infusion and selective condensation of silicon alkoxides within microphase-separated block copolymer templates dilated with scCO_2_ [31]. Depending on the ratios of TEOS and methyltriethoxysilane (MTES), it was possible to achieve different spatial pores packing: a cubic lattice of spheres at a zero MTES content, and a hexagonal packing of cylinders at an MTES content of 25–50%. A further increase in the MTES concentration leads to the disordered structure. The authors attributed this behavior to the nature of the precursors, which affects the hydrophobic–hydrophilic balance of the structure-forming agent. In particular, the authors explained the transition from packing of cylinders to packing of spheres by a change in the spatial nature of the microphase separation of the block copolymer template. 

**Figure 5:**
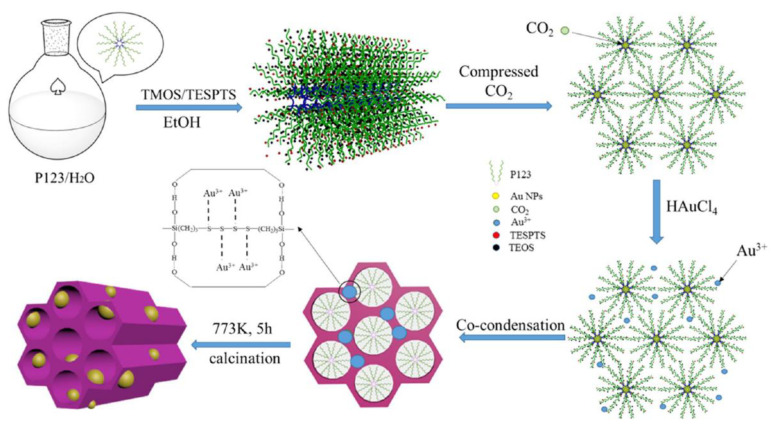
Schematic illustration of formation of the Au/mesoporous organosilicon composite with a one-pot approach. Reproduced with permission from Ref. [36]. Copyright 2018 American Chemical Society.

Systems of noble metal nanoparticles on various substrates are notable due to their promising optical, magnetic, and catalytic properties [32,33]. The key parameters are the dispersity of metal particles as well as the morphology of the resulting system as a whole. Changes in the mesostructure usually occur through variations of reactant ratios, medium pH, salt or organic additives introduction [34,35]. Using CO_2_ as the synthesis medium allows avoiding the classic problems associated with the necessity for post-processing, high cost, non-environmental technology, etc. In a recent work, gold nanoparticles deposited on oriented mesoporous organosilicon structures formed using compressed CO_2_ were obtained according to the “one-pot” scheme (Figure 5) [36]. The stages illustrated in the figure (with intermediate exposures) included: loading a structure-forming block copolymer into the reactor (Pluronic P123, water-alcohol solution), loading of organosilicon precursors (bis[3-(triethoxysilyl)propyl] tetrasulfide, tetramethoxysilane), CO_2_ pumping (pressure in the range of 2.9–5.9 MPa), subsequent loading of the gold precursor (HAuCl_4_, aqueous solution), drying and annealing (500 °C).

Variation of the CO_2_ pressure allowed a controlled transition from the tubular and hexagonal morphology of the formed substrate to the cellular and vesicular one. As it was described in [30], CO_2_ dissolved in the aqueous phase catalyzes the precursors hydrolysis, and its efficiency depends on pressure. Moreover, CO_2_ penetrates into the region of the hydrocarbon chains of the template, increasing the volume of the micelles and affecting the structure formation as a whole.

### 2.5. Composites Formed in scCO_2_

The high penetrating ability and diffusion rate of scCO_2_ are used for the synthesis of various types of composites, including those based on organosilicon compounds as well. The most common composites are network structures or dispersed particles of an inorganic phase incorporated into a polymer matrix. The benefits of using scCO_2_ to create such composites are described in detail in several reviews [5,37,38]; however, we will focus on some works not mentioned there.

Such composites are successfully used as various types of selective membranes and filters. The selectivity and overall efficiency of membranes largely depends on the accuracy of control of their characteristics, which is very difficult to achieve during synthesis. It is much easier to introduce particles with the required properties into the matrix in the second stage after the synthesis. In [39,40], the authors modified polymeric perfluorosulfonic acids (analogous to Nafion) by impregnation with a silane precursor and its subsequent conversion to polysiloxane using supercritical CO_2_. Since the modification with CO_2_ allows the new silicon-containing phase introduction along both hydrophilic ion channels and hydrophobic fluoropolymer domains, as well as their interfaces, it was possible to significantly suppress methanol permeability. Such microphase-separated membranes, in which nanosized proton-conducting channels are stabilized by the silicon-containing phase of the inclusions, can show better selectivity of ion transport in different power sources, reduced permeability for reagents (methanol, etc.), and also improved performance at elevated temperatures in fuel cells due to better retention of water.

Indeed, previously, our team described the classical process of silica composite obtaining with a polymer template: the TEOS silica precursor dissolved in scCO_2_ was delivered to the nanosized proton-conducting pores of Nafion matrix, where it was subsequently hydrolyzed and condensed in the presence of residual water due to the release of protons of sulfonic groups to catalyze the reaction [41]. It was possible to improve the thermomechanical properties and durability of the polymer and increase the hygroscopicity of the membrane, which led to enhanced and selective proton conductivity under low humidity conditions. The membranes with improved ion selectivity have shown promising performance in the operating cells of vanadium redox flow batteries.

## 3. Silicone Coatings, Silylation

In order to impart certain properties to the material, its surface can be coated with a layer of organosilicon compounds. The method of silylation from scCO_2_ is actively used primarily because of the possibility of uniform deposition of the material on substrates with complex surface geometry [42].

The main task of silylation is the functionalization of the material to create subsequently more durable organic–inorganic composite. The goal is to increase the affinity of the organic and inorganic phases to each other by forming a compatibilizing bifunctional layer at their interface.

The method of monolayer deposition of silanes on substrates made of hydroxyapatite, titanium dioxide, and hectorite from the supercritical CO_2_ phase was studied [43]. The obvious advantage of using scCO_2_ and silanes soluble in it is the possibility of creating homogeneous organosilicon coatings on hard-to-reach areas of the substrate such as deep and small pores, for example, of porous silicon oxide. Indeed, the approach was studied on model systems with different spatial pores scales (micro-, meso-, and macro-) [44].

The silanization of silicon substrates with an oxide layer is actively studied for microelectronics applications. Self-assembled layers of alkoxysilanes can be deposited on a silicon substrate to be used as barrier and adhesive layers in semiconductor devices [45].

In recent works, a group of French researchers studied the surface properties of a grafted alkoxysilanes layer on a silicon substrate with an oxidized surface layer [46]. By means of atomic force microscopy, it was possible to measure surface forces and to characterize the conformation of macromolecules in the obtained coatings. Depending on the nature of the precursor head group and the temperature of the system, various structures were formed during deposition: close-packed monolayers as well as inhomogeneous or dense bilayers. In the next study, the same group investigated the influence of the substrate morphology on the layer conformation: if in Ref. [46] alkoxysilanes were deposited on a flat sample of oxidized silicon, then in Ref. [47], the substrate consisted of planar silicon oxide nanochannels with a diameter of 3 and 5 nm. As a result, it was shown that the morphology of the coating is strongly influenced not only by the nature of the molecules but also by the geometry of the substrate.

Another type of research is devoted to the restoration of damaged surfaces of organosilicon materials by silylation from the scCO_2_ medium [48,49,50,51,52]. First of all, silylation is applied to restore porous dielectric materials with a low dielectric constant, which are required for the formation of new advanced semiconductor devices. The damages typically occur during the processing of such peculiar materials.

**Figure 6:**
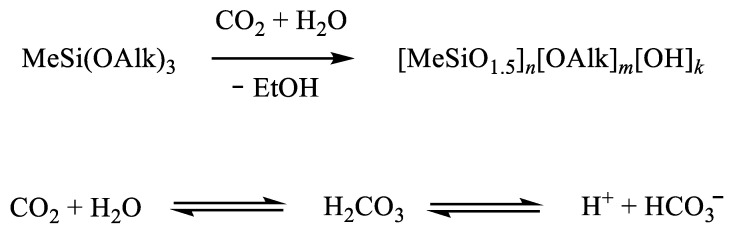
Scheme of hydrolytic polycondensation of methyltrialkoxysilane in the presence of carbonic acid. Adopted from Ref. [53].

In a similar aspect, it would be interesting to study the use of other silicon-containing agents in order to impart new properties to surfaces. It is known that hydroxyl-containing silsesquioxane oligomers can be obtained under pressure in water or in an aqueous phase saturated with carbon dioxide (i.e., in the presence of carbonic acid, Figure 6) [53]. The described low-molecular-weight soluble polymethylsilsesquioxanes of various structures can act as materials for the formation of antiabrasive coatings due to high adhesion to a wide range of polar substrates provided by the presence of a large number of hydroxyl groups.

The study of the deposition of such silanes in CO_2_ is a promising direction and requires further development.

## 4. PDMS Role in Pore Nucleation Process during Decompression

The affinity of some organosilicon compounds to CO_2_, in particular, polydimethylsiloxanes, makes it possible to use them to increase the swelling degree of polymer matrices in CO_2_. PDMS in CO_2_ medium plays the role of an accumulator of the fluid molecules. It was shown that these properties make it possible to vary the process of pore formation in polystyrene polymer matrices with the addition of PDMS, which promotes nucleation upon decompression [54,55].

The addition of PDMS to the CO_2_/polystyrene system increases both the diffusion coefficient of the fluid molecules (by two times) and the swelling degree of the polystyrene/PDMS composite [56]. A high initial concentration of CO_2_ in the polymer during decompression and high velocities of fluid molecules lead to a larger volume of the formed porous structure. By varying the molecular lengths of PDMS additives, it was found that the usage of siloxanes with M_n_ ≈2000 resulted in a 40% reduction in pore size with a simultaneous increase in the volume expansion ratio as compared to polystyrene foamed without additives.

Similar studies were carried out for a polypropylene (PP) matrix [57]. Due to the ability of PP to crystallize, the pore formation with the addition of PDMS leads to a decrease in the CO_2_ desorption rate. The affinity of PDMS to CO_2_, as well as its role as a nucleation promoter, leads to smaller and more uniform pores formed during decompression.

Of great interest is the production of highly porous foams based on PDMS itself, which are used in an extremely wide range of applications: from the water purification from hydrophobic impurities tasks, to biomedical devices, flexible microfluidic devices, electronic skin for prostheses and robots (bionic system designed to imitate some physiological properties of human skin), epidermal electronics, highly sensitive capacitive pressure sensors, etc. [58,59,60,61]. Porous structures are most often obtained by coating a PDMS layer on different porous templates, which is followed by removal of the substrate. In particular, an easy-to-implement method is the preparation of a sponge-like PDMS structure using sugar particles as a porogen [62].

A demanding task seems to be creating the required porous morphology of materials based on PDMS by inducing phase separation in supercritical CO_2_ medium. In relation to PDMS, this has not yet been studied. Nevertheless, such a well-known approach [63,64] makes it quite easy to control the degree of material porosity in the range of 30–60%, which was shown for the films of some other polymers [65].

## 5. Polysiloxanes as Polymerization Stabilizers

Siloxane oligomers are widely used as surfactants, including for conventional (performed at normal pressure) emulsion and dispersion polymerizations [66,67,68]. They replaced stabilizers based on polyhydroxystearic acid, which requires the introduction of additional organic solvents for effective stabilization [68].

Recently, polymerization in supercritical CO_2_ has been widely used. Dispersion polymerization in scCO_2_ medium is a process when soluble in scCO_2_ substances are used as monomers and initiators. Here, the resulting polymer loses solubility in the fluid when a certain molecular weight is achieved. In order to prolong the growth of polymer chains and prevent premature coagulation and precipitation, special amphiphilic additives affinitive both for CO_2_ and polymer are used. In general, the use of scCO_2_ as a medium for dispersion polymerization has already been described in some reviews [69,70].

This approach makes it possible to carry out the synthesis using a small amount of organic solvents and to minimize the stages of post-treatment and disposal. The main disadvantage of using CO_2_ medium was the narrow range of particle size control. However, this problem was also partially solved by varying the synthesis parameters (variation of the monomer and stabilizer loading, stepwise introduction of the monomer) [71].

The first work on the use of siloxanes as an amphiphilic stabilizer and CO_2_-soluble macromonomer was published in 1996 [72]. The authors justify the choice in favor of siloxanes, in comparison with fluoropolymers, both by the high cost of the latter and by the fact that siloxanes are soluble in a large number of common solvents, which simplifies further study of the obtained polymers.

The use of PDMS and PDMS-based macromolecules as stabilizers of polymerization reactions in scCO_2_ is actively applied to solve various problems of polymer synthesis. Poly(methyl methacrylate) can be obtained using vinyl-terminated PDMS chains [73] and methacrylate-terminated monomethacrylate–PDMS macromonomer [74] as stabilizers. At the same time, varying the system parameters, including the reagents concentration, makes it possible to change the morphology of the resulting latex particles. By varying the monomer and stabilizer loadings, it was possible to obtain poly(methyl methacrylate) particles in a wide size range from 0.5 to 5 μm [71].

PDMS homopolymer is also used for the polymerization of phenol/furfural gel microspheres [75], and its vinyl-terminated modification is used for the in situ implementation of Copaiba balsam in growing poly(methyl methacrylate) particles for pharmaceutical applications [76]. A triblock copolymer based on PDMS and polycaprolactone is widely used for L-lactide polymerization [77]. The dispersion polymerization of glycidyl methacrylate in scCO_2_ was successfully carried out using the monomethacrylate-PDMS macromonomer [78].

Supercritical CO_2_ itself is a convenient medium for the synthesis of more complex stabilizers based on PDMS. In Ref. [79], the researchers synthesized similar stabilizers: triblock copolymers based on hydroxypropyl-terminated PDMS as the CO_2_-philic part and poly(L-lactide) as the hydrophilic part of the stabilizer.

Since the pioneering use of PDMS-based stabilizers for polymerization in scCO_2_ [72], the proposed approach has been actively developed, which is confirmed by dozens of new published studies. PDMS and stabilizers based on it are optimal for use in supercritical CO_2_ from both economic and chemical points of view and are promising for further study.

## 6. Extraction and Fractionation in scCO_2_

One of the most obvious and studied ways of using supercritical fluids, including scCO_2_, is extraction. Extraction is the process of dissolving a compound or a group of compounds from some matrix into an external solution using a supercritical fluid as an eluent. The main features of scCO_2_, such as the absence of capillary effects and high dissolving power, with the possibility of its control, allow the fluid to penetrate the entire volume of the substrate, including those with complex morphology, and extract a wide class of substances from it. The subsequent reduction in pressure of the supercritical fluid allows the dissolved extract to precipitate and separate from the eluent, which turns into a gas. The gaseous state of CO_2_ at normal pressure eliminates the problem of residual solvent in both the extract and the purified matrix.

The safety and nontoxicity of scCO_2_ primarily determine its frequent use for food industry tasks: this includes the extraction of various oils, flavonoids, vitamins, and other compounds, which is described in a large number of both original works and reviews [80,81,82,83]. For the same reason, scCO_2_ is also ubiquitously used for pharmaceutical purposes [84].

Despite the fact that the most large-scale polymers are not soluble in scCO_2_, it can be useful in the preliminary extraction of certain introduced additives in order to reuse them or to simplify the processing of the polymers themselves. These can be various dyes, plasticizers, modifiers, additives, stabilizers, flame retardants, coupling agents, antioxidants, residual monomers, oligomers, initiators, catalysts, pollutants, and other substances [85]. There are works on the extraction of functional additives, including antioxidants, from polypropylene and polyethylene matrices using supercritical fluids [86,87,88].

The extraction is of particular importance to the production of CO_2_-soluble polymers: the isolation of low-molecular-weight fraction from the resulting product should significantly increase its monodispersity, and hence the quality and cost.

The first work on PDMS fractionation in scCO_2_ by Yilgör and McGrath was published in 1984 [89]. It describes a method for the separation of PDMS oligomers with respect to their chain length into six fractions with a narrow dispersion: the achieved polydispersity index (PDI = M_w_/M_n_) was in the range of 1.1–1.4. The number average molecular weights of the oligomers to be separated were 2000 and 6400 g mol^−1^, while the measurement of the molecular weights of the fractionated parts was carried out by potentiometric titration. At the same time, since the first isolated fraction consists of the lowest molecular weight cyclic siloxanes due to the equilibrium nature of the reaction for obtaining PDMS [90], its titration is impossible due to the absence of titratable groups (COOH).

**Figure 7:**
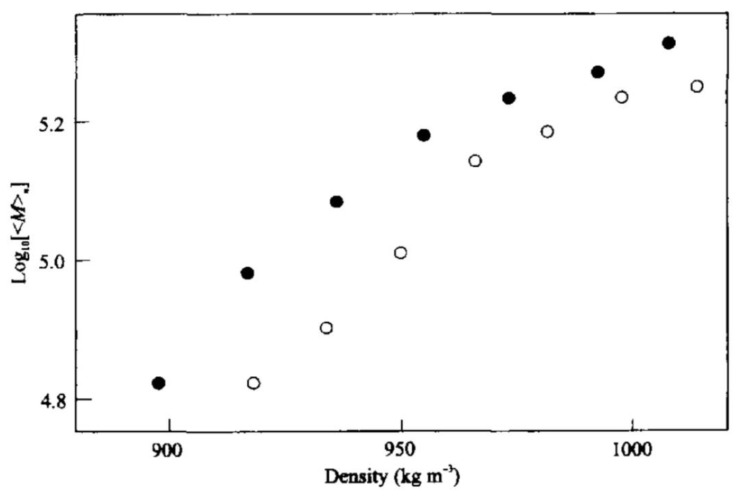
Supercritical extraction of polydimethylsiloxane using a linear density program; the graph shows the logarithm of the number-averaged molar masses of the fractions versus fluid density with filled circles (●) for a lower rate of density increase and open circles (○) for a higher rate. Reproduced from Ref. [91], Copyright (1997), with permission from Elsevier.

Numerical simulation, as well as experimental study, were conducted and compared for fractioning process of PDMS and other compounds using a supercritical fluid (Figure 7) [91]. The extraction was carried out at 40°C in the range of 260–570 bar, and it was possible to separate seven fractions of PDMS with M_n_ in the range of (60–200) 10^3^ g mol^−1^.

**Figure 8:**
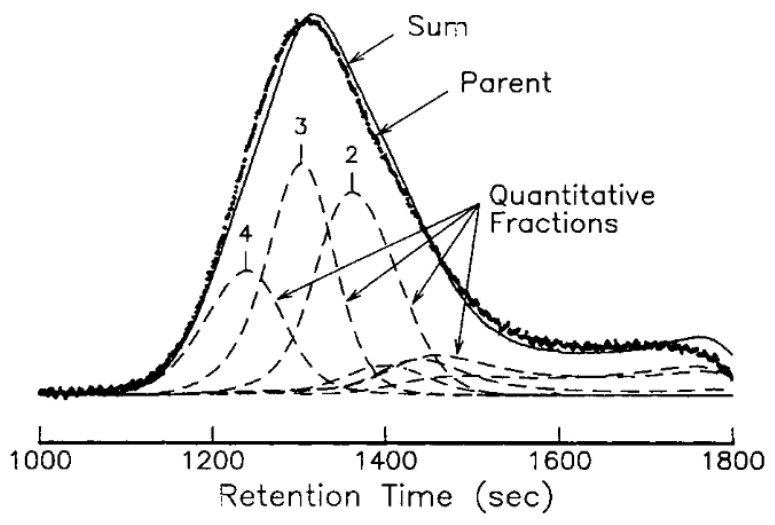
Gel permeation chromatogram of stock and fractionated PDMS samples separated at 70 °C. Reproduced with permission from Ref [93], copyright 1995 John Wiley and Sons.

Another group of authors carried out PDMS fractionation in the M_n_ range from 3 × 10^3^ to 30 × 10^3^ g mol^−1^ in the PDMS–toluene–CO_2_ ternary system [92]. At a temperature of 40 °C and a pressure of 130 to 210 bar, the polydispersity of the resulting fractions ranged from 1.4 to 2.0. In another work, at various temperatures in the range of 60–90 °C, it was possible to separate PDMS fractions with M_n_ from 40 to 240 × 10^3^ g mol^−1^ (Figure 8) [93].

The phase behavior of the PDMS/CO_2_ binary system was studied in a practically important range of temperatures and pressures. Thus, the phase diagrams of the hydroxyl-terminated PDMS system and CO_2_ were obtained at temperatures of 40 °C, 50 °C, 60 °C and a polymer molecular weight of 2750 and 18,000 g mol^−1^ (PDI = 1.59 and 1.29, respectively) [94]. Data for a conventional PDMS/CO_2_ system in the temperature range from 30 to 190 °C and polymer mass in the range from 40 × 10^3^ to 370 × 10^3^ g mol^−1^ (PDI in the range from 2.2 to 3) are presented in [95,96].

The solubility of PDMS in scCO_2_ opens up a wide range of possibilities in its production process in comparison with almost any other polymers. One of the main problems of the large-scale production of PDMS is the purification of the obtained material from the low-molecular-weight fraction, including oligomers and monomers, which can be carried out by means of supercritical extraction. This requires detailed studies of the phase behavior of the PDMS/CO_2_ system in a wide range of polymer lengths and macroparameters.

## 7. Conclusions

Compared to conventional liquid solvents, supercritical fluids provide faster diffusion, reduced viscosity, and the ability to separate products, residual reactants, and catalysts for subsequent reuse. In particular, the fast reaction kinetics, the absence of capillary forces as well residual solvent problem, and environmental safety lead to the expansion of supercritical CO_2_ usage for organosilicon compounds as well. Due to their convenience and low cost, much attention is paid to the use of polydimethylsiloxanes as stabilizers for dispersion polymerization in scCO_2_, silylation, and pore formation. The extraction of organosilicon compounds was described in relatively early works, and many technological processes were then successfully implemented in industry. The performed analysis shows that this field still retains a great research potential for development both in terms of such technological operations as the surface treatment of various materials and products (textiles, implants, electronic devices, porous materials) and in relation to chemical transformations using reactive silicone oligomers of various structures.

## Data Availability

The data presented in this study are available on request from the corresponding author.
